# A Rare Case of a Transgender Female With Breast Implant–Associated Anaplastic Large Cell Lymphoma Treated With Radiotherapy and a Review of the Literature

**DOI:** 10.1177/2324709619842192

**Published:** 2019-04-22

**Authors:** Naba Ali, Kunal Sindhu, Richard L. Bakst

**Affiliations:** 1 University of Rochester, Rochester, NY, USA; 2 Mount Sinai Medical Center, New York, NY, USA

**Keywords:** BIA-ALCL, radiotherapy, transgender

## Abstract

We are reporting a case of a 54-year-old transgender female with a history of breast augmentation with bilateral silicone implants. Seventeen years later, she presented with an enlarging right breast mass. Pathology confirmed breast implant–associated anaplastic large cell lymphoma (Ann Arbor Stage IIE, TNM Stage III BIA-ALCL). The patient underwent bilateral capsulectomy, sentinel lymph node biopsy with adjuvant CHOP (cyclophosphamide, doxorubicin, vincristine, and prednisone) chemotherapy, and radiotherapy to the right chest, axilla, and supraclavicular lymph nodes. BIA-ALCL is a rare entity, especially in transgender females. We report this case and a review of the literature in this report.

## Introduction

Breast implant–associated anaplastic large cell lymphoma (BIA-ALCL) is a rare subtype of T-cell non-Hodgkin’s lymphoma, which occurs in the capsule or periprosthetic fluid surrounding a breast implant placed for reconstructive or cosmetic indications. It is a subtype of anaplastic large cell lymphoma that is CD30 positive and ALK negative. Morphologically, the tumor consists of large pleomorphic lymphoid cells with horseshoe-shaped nuclei and abundant cytoplasm.^1,2^

The first case was reported in 1997 by Keech and Creech with a saline-filled breast implant.^3^ Since then, more than 300 cases of BIA-ALCL have been reported in the literature, and a retrospective study estimated the lifetime prevalence of BIA-ALCL in the United States as 1 per 30 000 patients with textured breast implants.^4^ However, only 3 cases have been reported in transgender male to female patients.^5-7^ In this article, we describe another rare case of invasive BIA-ALCL in a transgender female.

## Case Presentation

A 54-year-old transgender African American female with a history of bilateral breast augmentation presented to our clinic with a long history of right breast discomfort. She began hormonal therapy in 1987, and socially transitioned from male to female in 1990. In 2000, she underwent breast augmentation surgery, receiving bilateral silicone implants. In 2009, she developed pruritus and hyperpigmentation of the skin overlying her right breast but did not seek medical care. Several years later, she noticed an enlarging mass in her right breast. After acquiring health insurance, she presented to her primary care physician in December 2017 to discuss further care.

Physical examination at that time revealed a 1.5 cm nontender, fixed right breast mass with overlying hyperpigmented skin. Mammogram and right breast ultrasound in January 2018 showed a suspicious breast mass encasing the right implant at 4:30, 7 cm from the nipple (Breast Imaging Reporting and Data System [BIRADS]-4). Ultrasound-guided right breast biopsy revealed atypical T-cells positive for CD30, EMA, and CD2, and negative for CD3, CD43, CD20, and PAX5. The findings were consistent with BIA-ALCL. Biopsy of the hyperpigmented area was benign, consistent with seborrheic keratosis.

**Figure 1.**
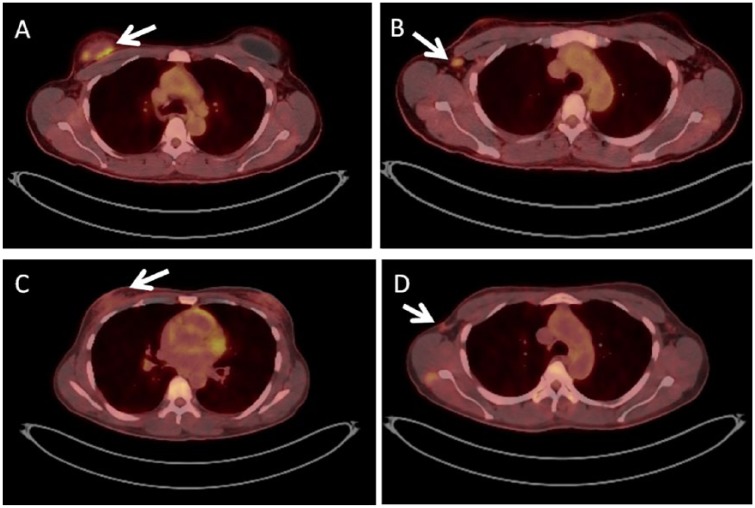
Axial PET scans. A and B were acquired prior to chemotherapy, while C and D were acquired after 6 cycles of CHOP. (A) Demonstrates hypermetabolic soft tissue densities around the breast implant, while (B) shows a hypermetabolic right axillary lymph node. (C and D) Show residual FDG-avidity in the right chest and axilla, respectively.

An initial positron emission tomography/computed tomography scan (PET/CT; Figure 1) demonstrated 4 abnormal hypermetabolic soft tissue densities surrounding the right breast implant (SUV [standardized uptake value] maximum 4.8) and a 1.3 × 0.5 cm hypermetabolic enlarged right axillary lymph node (SUV maximum 3.2). Though core needle biopsy of the right axillary lymph node was insufficient for diagnosis, she was presumed to have Ann Arbor Stage IIE, TNM Stage III BIA-ALCL.

The patient subsequently underwent bilateral breast implant removal, capsulectomy, and sentinel lymph node biopsy. Surgical pathology revealed BIA-ALCL inside and outside of the right breast capsule, 2/2 right sentinel axillary lymph nodes positive for BIA-ALCL, and benign skin of the left and right breast. The patient was then presented to the multi-disciplinary tumor board at our institution, which recommended that she receive adjuvant chemotherapy and/or radiotherapy.

The patient received 4 cycles of cyclophosphamide, doxorubicin, vincristine, and prednisone (CHOP) before undergoing repeat PET/CT, which showed a favorable response to treatment as evidenced by an interval decrease in the FDG (fluorodeoxyglucose)-avid soft tissue foci in the right breast (SUV maximum 2.4) and no hypermetabolic lymphadenopathy. She then received 2 more cycles of CHOP. Post-chemotherapy PET/CT (Figure 1) showed FDG-avidity in the right axilla (SUV maximum 2.4) and right chest (SUV maximum 2.1).

**Figure 2.**
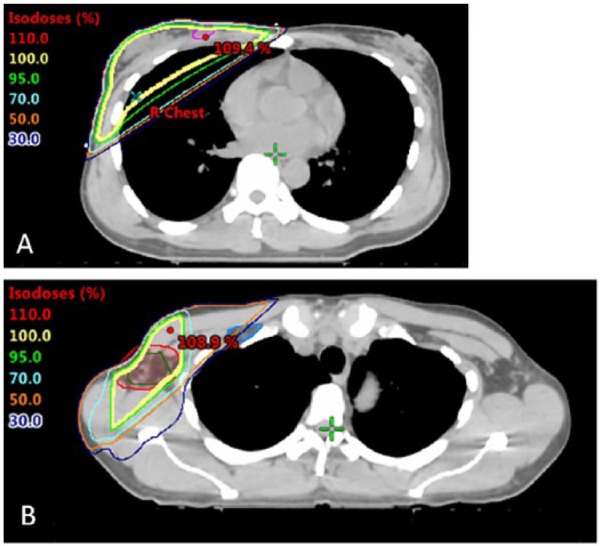
Radiation treatment plans: (A) Right chest, axilla, and supraclavicular lymph node plan (3000 cGy) and (B) Cone down to the right axilla (600 cGy).

Following chemotherapy, the patient went on to receive adjuvant radiation therapy. She received 3000 cGy over 15 fractions to the right chest, right axilla, and right supraclavicular lymph nodes, followed by a cone down consisting of 600 cGy in 3 fractions delivered to the right axilla (Figure 2). Her treatment was delivered utilizing 3-dimensional conformal technique. She tolerated the treatment well without any difficulties.

## Discussion

BIA-ALCL is a rare T-cell lymphoma that commonly presents with spontaneous fluid collection or enlarging capsular mass 8 to 10 years following implantation of a breast implant. However, patients may present at an earlier or later time, as in this case. The etiology of this disease is largely unknown. However, the majority of cases demonstrate an association with use of a textured implant.^8-10^ Breast implants can be separated into 2 groups based on the surface of their shell: textured or smooth. The textured surface is rough and provides traction with the surface of the breast pocket, aiding in the stabilization of the implant and preventing capsular contracture. Alternatively, the smooth surface is sleek and slippery, providing a more natural physical shape. Cases have been identified in patients with both saline- and silicone-filled implants. The Food and Drug Administration reported that of the 272 medical device reports received with information regarding implant surfaces, 242 implants had textured surfaces. Additionally, among 413 reports with information regarding implant fill types, 234 reported a silicone gel filling and 179 reported a saline filling.^11^

Furthermore, while the incidence of primary BIA-ALCL in the general US population is low, women with textured implants have a significantly higher incidence as evidenced by a retrospective study of 100 pathologically confirmed BIA-ALCL cases in the United States demonstrating a 67.6-fold higher incidence rate.^4^ Evidence suggests that BIA-ALCL develops in the setting of chronic inflammation likely induced by the textured implant,^12^ and pathologic examination of prior cases has identified extensive lymphoid neogenesis adjacent to the fibrous capsule.^13^

The majority of data regarding treatment of BIA-ALCL is based on case reports and case series due to the rarity of the disease. The National Comprehensive Cancer Network (NCCN) has suggested standardized guidelines based on this evidence. For all patients with BIA-ALCL, complete surgical resection is recommended. This involves implant removal, total capsulectomy, and negative tissue margins surrounding any disease mass. For patients with localized disease that can be completely excised, no adjuvant therapy is recommended. However, for patients with extended disease and regional lymph node involvement, adjuvant chemotherapy with an anthracycline-based regimen or brentuximab vedotin is recommended.^14^ The role of radiation therapy in the treatment of BIA-ALCL remains unclear. However, NCCN guidelines suggest that it may provide a benefit for patients with local residual disease, positive margins, or unresectable disease with chest wall invasion.^14^

There are currently only 3 reports of BIA-ALCL in transgender women. All 3 patients presented as Ann Arbor Stage IE and underwent treatment with complete surgical resection. Two of these patients also underwent adjuvant CHOP chemotherapy. All 3 cases occurred in association with textured implants, 2 of which were silicone gel filled and 1 unreported.^5-7^ Table 1 summarizes relevant information from these studies. Here, we have presented the first case of a transgender woman with Ann Arbor Stage IIE disease. Additionally, this is the first case of a transgender female with BIA-ALCL treated with radiation therapy. Several case reports of cisgender women with Stage IIE disease have seen clinical and radiographic remission with radiation doses of 3000 to 4000 cGy in 180 cGy fractions.^15-17^ Thus, we elected to use a dose of 3600 cGy.

**Table 1.** Summary of Prior Case Reports of Transgender Males to Females With BIA-ALCL.

Patient	Study	Year of Publication	Initial Breast Surgery	Interval to Presentation (Years)	Presentation	Histopathology	Imaging	Ann Arbor Stage	Treatment
1	Orofino et al^5^	2016	Bilateral breast augmentation in 2007. Revision surgery (explantation and implantation) with textured implants (Allergan) in 2008. Left implant definitively removed in 2010.	7	Presented with diffuse pruritus and cutaneous papules, mild leukocytosis, hyper eosinophilia, and elevated LDH.	Histological examination of the pseudo-capsule showed the diffuse infiltration of large cells with irregular, anaplastic or “embryo-like” nuclei and abundant eosinophilic granular cytoplasm. Malignant population was CD30+ and ALK-1 negative.	PET/CT revealed uptake in left breast (SUV 9.5). MRI revealed subcutaneous effusion and a 7 × 12 cm mass without contrast enhancement.	Stage IE	Radical left mastectomy and 4 cycles of adjuvant CHOP chemotherapy.
2	de Boer et al^7^	2017	Bilateral breast augmentation with Nagor GFX silicone-filled textured implants in 1998 followed by 3 revision surgeries (explantation and implantation) in 1999, 2012, and 2015.	20	Rapidly enlarging left breast. “Late-onset” periprosthetic seroma of the left breast.	Examination of capsule demonstrated a small collection of atypical lymphoid cells adherent to the inner surface of the fibrous capsule. No infiltrating component observed. Large atypical lymphoid cells were abundant in the seroma fluid. Malignant population were CD30+ and ALK-1 negative.	Ultrasonography demonstrated seroma surrounding periprosthetic space.	Stage IE	Bilateral explantation and capsulectomy. No chemotherapy or radiation.
3	Patzelt et al^6^	2018	Bilateral breast augmentation in 2007 with silicone gel filled textured implants (Allergan) in Czech Republic.	7	5-cm tumorous mass in her left breast.	Examination of the capsule demonstrated tumor cells with vesicular nuclei and prominent nucleoli, disco hectically organized. Malignant population was CD30+ and ALK-1 negative.	MRI revealed a ruptured implant and a tumorous mass penetrating into the capsule and infiltrating the pectoral muscle.	Stage IE	Explantation and capsulectomy of left breast implant. Six cycles of CHOP-21. Tumor free at 2-year follow-up.

Abbreviations: BIA-ALCL, breast implant–associated anaplastic large cell lymphoma; PET/CT, positron emission computed tomography; SUV, standardized uptake value; MRI, magnetic resonance imaging; CHOP, cyclophosphamide, doxorubicin, vincristine, and prednisone; LDH, lactate dehydrogenase.

In conclusion, BIA-ALCL is a very rare lymphoma associated with textured implants. This case suggests that BIA-ALCL can occur in transgender males to females, further illuminating the population at risk for the development of this disease. Thus, all patients, including transgender females, should be provided education regarding this risk and monitored for development of disease. Patients can be effectively treated with surgical resection and consideration of adjuvant chemoradiation therapy, according to NCCN guidelines. We suggest a radiation dose of 3600 cGy in 180 cGy fractions.
